# Editorial: Plants as Alternative Hosts for Human and Animal Pathogens – Second Edition

**DOI:** 10.3389/fmicb.2020.01439

**Published:** 2020-07-14

**Authors:** Adam Schikora, Robert Wilson Jackson, Leo Van Overbeek, Nicola Holden

**Affiliations:** ^1^Julius Kühn-Institut, Institute for Epidemiology and Pathogen Diagnostic, Braunschweig, Germany; ^2^School of Biosciences and Birmingham Institute of Forest Research, University of Birmingham, Birmingham, United Kingdom; ^3^Wageningen Plant Research, Wageningen University and Research, Wageningen, Netherlands; ^4^The James Hutton Institute, Cell and Molecular Sciences, Dundee, United Kingdom

**Keywords:** human pathogens on plants, *Salmonella*, *E. coil*, fresh produce, crop plants

Outbreaks of food-borne diseases are often associated with minimally processed fruits and vegetables. Interactions between human pathogens and plants are not incidental, transient or passive. For many, there is a defined and specific molecular basis. Both partners, the microbe and plant, play active roles in this interaction. Our understanding on how human pathogens colonize plants, especially in the face of microbial competition, is far from clear. Since these pathogens pose a health risk, important aspects include detection and surveillance, inevitably related to epidemiology, risk analysis, and therefore relevant to food safety, whether in the field or in the factory. This eBook second edition provides new discoveries in the field and incorporates a collection of articles focusing on several aspects of interactions between diverse human pathogens and crop plants. Those range from comprehensive approaches in food safety, through adaptation to the plant environment to shifts in the structure of microbial communities during the change in global climate.

Requirement for safe produce is of utmost importance for consumers and producers. Since it is not possible to remove microbes from leafy green vegetable production, avoidance of contamination with human pathogens such as Shiga toxin-producing *Escherichia coli* (STEC), *Salmonella enterica, Shigella, Yersinia enterocolitica*, or *Listeria monocytogenes*, is crucial. These have the potential to lower the risk of contamination. How such critical points can be combined to decrease risks and improve microbial stability, nutritional properties and quality of leafy vegetables, is a key question. A toolbox with different physical, physiochemical, and microbial possibilities could be an option (Mogren et al.). The authors present mechanisms, which could be used for leafy greens and other produce. To enter food production systems, human pathogens need to adapt and establish in an environment, which is not optimal for their survival. Those environments, for example soil or plant tissues, have a significant impact on their physiology and influence additionally the virulence toward the plant host. The effects of soil type, organic fertilization, and plant species on the survival of *S. enterica* in soil as well as the colonization of plants are very substantial (Jechalke et al.). Authors revealed that different strains of *S. enterica* were able to persist in soil for several weeks and colonize leaves of lettuce and corn salad. Moreover, *S*. Typhimurium 14028s responded to lettuce or lettuce root exudates showing an upregulation of genes associated with biofilm formation and virulence. In addition to *Salmonella, E. coli* O157:H7 responds to physicochemical stresses in cut lettuce and lettuce lysates by upregulation of several stress response pathways. Damaged lettuce leaf tissue supplied *E. coli* with substrates for proliferation, and also provides pathogenic bacteria with choline for osmotic protection (Scott et al.). Internalization of bacterial cells into stomata enables human pathogens to evade the hostile environment of the leaf surface and reside in a protected, nutrient-rich niche. However, growth condition, different preadaptation, and temperature have an impact on the internalization abilities of *Salmonella enterica* (Kroupitski et al.). The authors further emphasized the function of universal stress-related genes in leaf internalization, highlighting the complexity of the bacterial internalization process. The above studies clearly demonstrated the ability of human pathogenic bacteria to adapt to the plant environment. How this adaptation differs from the adaptation to animal hosts is a very intriguing question. Comparison of the metabolic pathways, induced in those different environments, revealed that biosynthesis of amino acids, purines, vitamins, and the catabolism of glycerol and glucose are important (Kwan et al.). Nevertheless, whereas biosynthesis and degradation of fatty acid contributed to *S. enterica* animal colonization, it seems that only fatty acid biosynthesis was required during plant colonization. A genome-wide mutant screen comparing the plant-associated *S*. Newport with the animal-associated *S*. Typhimurium emphasized the importance of amino acid biosynthetic pathways and iron acquisition during the persistence in tomatoes (de Moraes et al.). Besides, a newly discovered gene, *papA*, which is unique to *S*. Newport contributes to its fitness in tomatoes. Homologs of *papA* are present in bacteria that colonize both plants and animals. Human pathogens do not restrict themselves to reaction and adaptation to a plant environment. They produce plant hormones, which may influence their vegetal host. The *Salmonella ipdC* gene, responsible for production of auxin via the IPyA pathway, is expressed on root surfaces, increasing the formation of lateral roots. Very interestingly, the *ipdC* mutant is less virulent in a murine model, because of a defect in its ability to cross the intestinal barrier (Cox et al.). Yet another aspect important in plant production systems is the occurrence of insects. The presence of leafhoppers results in longer persistence of *S. enterica* in plants. The reason could be the activation of contrary signaling pathways in the plants, whereby leafhopper infestation activated jasmonic acid defense responses while *S. enterica* colonization triggered salicylic acid responses. Such a situation hinders the defense ability of the plant (Cowles et al.). In addition to bacteria, also pathogenic fungi inhabit plant production systems. Species from the *Fusarium solani* complex are frequently isolated from soils. They are one of the major opportunistic human pathogenic filamentous fungi, responsible, for example, for mycotic keratitis. Unfortunately, first-generation treatments, like triazoles, fluconazole, or itraconazole, are already ineffective. However, despite the intensive use of azoles, fusaria have not developed resistance against imidazole, leaving a treatment opportunity (Homa et al.). Global warming represents another challenge for the quality and safety of produce. Long-term analysis of moderate surface warming revealed a reduction of beneficial bacteria and an increased presence of potential pathogenic bacteria in the phyllosphere of plants. Such changes bear the risk for transmission of both plant and human pathogens in plant production systems (Aydogan et al.).

It is clear that interactions between human pathogens and plants are still not fully understood. Therefore, a collective effort is needed in order to develop new approaches and strategies in plant and consumer protection.

This Research Topic aligns closely with the networking partnership of the European COST Action 16110 “Control of Human Pathogenic Micro-organisms in Plant Production Systems” (www.huplantcontrol.igzev.de).

## Author Contributions

All authors listed have made a substantial, direct and intellectual contribution to the work, and approved it for publication.

## Conflict of Interest

The authors declare that the research was conducted in the absence of any commercial or financial relationships that could be construed as a potential conflict of interest.

